# A Mathematical Model for MicroRNA in Lung Cancer

**DOI:** 10.1371/journal.pone.0053663

**Published:** 2013-01-24

**Authors:** Hye-Won Kang, Melissa Crawford, Muller Fabbri, Gerard Nuovo, Michela Garofalo, S. Patrick Nana-Sinkam, Avner Friedman

**Affiliations:** 1 Mathematical Biosciences Institute, Ohio State University, Columbus, Ohio, United States of America; 2 Davis Heart and Lung Research Institute, Ohio State University, Columbus, Ohio, United States of America; 3 Department of Molecular Virology, Immunology and Medical Genetics, Ohio State University, Columbus, Ohio, United States of America; 4 Department of Mathematics, Ohio State University, Columbus, Ohio, United States of America; University of Edinburgh, United Kingdom

## Abstract

Lung cancer is the leading cause of cancer-related deaths worldwide. Lack of early detection and limited options for targeted therapies are both contributing factors to the dismal statistics observed in lung cancer. Thus, advances in both of these areas are likely to lead to improved outcomes. MicroRNAs (miRs or miRNAs) represent a class of non-coding RNAs that have the capacity for gene regulation and may serve as both diagnostic and prognostic biomarkers in lung cancer. Abnormal expression patterns for several miRNAs have been identified in lung cancers. Specifically, let-7 and miR-9 are deregulated in both lung cancers and other solid malignancies. In this paper, we construct a mathematical model that integrates let-7 and miR-9 expression into a signaling pathway to generate an in silico model for the process of epithelial mesenchymal transition (EMT). Simulations of the model demonstrate that EGFR and Ras mutations in non-small cell lung cancers (NSCLC), which lead to the process of EMT, result in miR-9 upregulation and let-7 suppression, and this process is somewhat robust against random input into miR-9 and more strongly robust against random input into let-7. We elected to validate our model in vitro by testing the effects of EGFR inhibition on downstream MYC, miR-9 and let-7a expression. Interestingly, in an EGFR mutated lung cancer cell line, treatment with an EGFR inhibitor (Gefitinib) resulted in a concentration specific reduction in c-MYC and miR-9 expression while not changing let-7a expression. Our mathematical model explains the signaling link among EGFR, MYC, and miR-9, but not let-7. However, very little is presently known about factors that regulate let-7. It is quite possible that when such regulating factors become known and integrated into our model, they will further support our mathematical model.

## Introduction

Lung cancer is the leading cause of cancer-related deaths worldwide. In the U.S. the number of new occurrences is approximately 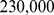 annually, and the number of deaths is 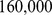, representing 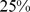 of all cancer related deaths [1]. Lack of early detection and limited options for target therapies are both contributing factors to the dismal statistics observed in lung cancer. Thus, advances in both of these areas are likely to lead to improved outcomes.

microRNAs (miRs or miRNAs) represent a class of non-coding RNAs that have the capacity for gene regulation and may serve as diagnostic and prognostic biomarkers in lung cancer. Abnormal expression patterns for miRNAs have been identified in lung cancers. Specifically, let-7 and miR-9 are deregulated in both lung cancers and other solid malignancies. Takamizawa et al. (2004) and Nicoloso et al. (2009) demonstrated that let-7 is downregulated in non-small cell lung cancers (NSCLC) [2], [3]. Several investigators have shown that let-7 harbors tumor suppressive properties both in vitro and in vivo [4], [5]. Using microarray data, Yanaihara et al. (2006) reported that miR-9 was decreased in NSCLC [6], whereas Volinia et al. (2006) reported an increase in miR-9 expression [7]. More recently Crawford et al. (2009) reported increased expression of miR-9 in NSCLC [8], and Võsa et al. (2011) drew the same conclusion from their microarray data [9]. Recently, we have also independently analyzed 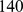 cases of NSCLC and compared miR-9 expression between tumors and adjacent uninvolved lung tissue. We found that in approximately 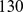 cases miR-9 was overexpressed in lung tumors; see Supplementary Material S1. A recent investigation showed that miR-9 contributes to metastatic potential in breast cancer in part by targeting components of epithelial mesenchymal transition (EMT) [10]. However, the role for miR-9 in the pathogenesis of lung cancer is less well understood. Mascaux et al. (2009) demonstrated an induction in miR-9 expression during bronchial squamous carcinogenesis [11].

Given the fact that a single miRNA may regulate tens to hundreds of genes, understanding the importance of an individual miRNA in cancer biology can be challenging. This is further complicated by observations that the dysregulation of several miRNAs is often required to cause a given phenotype. To date, few models exist to elucidate the mechanisms by which multiple miRNAs contribute both individually and in tandem to promote tumor initiation and progression. Applying mathematical modeling to miRNA biology provides an opportunity to understand these complex relationships. In the current study, we have developed for the first time a mathematical model focusing on miRNAs (miR-9 and let-7) in the context of lung cancer as a model system; however, our model system could be applicable to miRNA biology in both malignant and benign diseases. For simplicity, we have integrated these miRNAs into a signaling pathway to generate an in silico model for the process of EMT. Herein, we include the EGF-EGFR complex and associated downstream signaling culminating in matrix metalloproteinase (MMP) expression. Other components of our pathway include SOS, Ras, ERK, MYC,E-Cadherin, miR-9, and let-7.

We have simulated the model under several scenarios of gene mutations that may lead to lung cancer and determined, in each scenario, that miR-9 was upregulated and let-7 downregulated. We have also shown that the process leading to EMT is somewhat robust against random input into miR-9 and more strongly robust against random input into let-7.

## Results

### Biological Background

**Figure 1:**
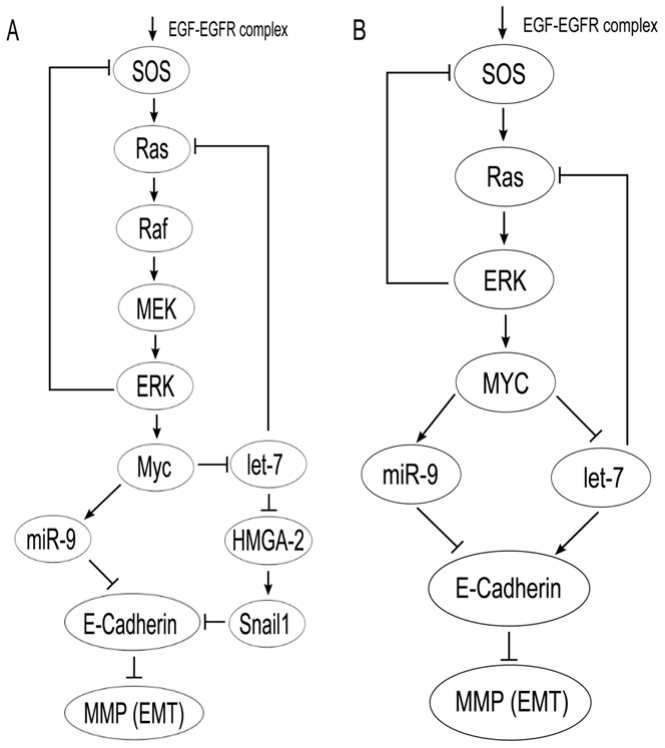
A signaling pathway for lung cancer. A pathway from EGF-EGFR complex to MMP, which includes miR-9 and let-7, is given in (A) and a simplified pathway is shown in (B).

Figure 1 A shows a signaling pathway involving miR-9, let-7, MYC, and EMT, while Figure 1 B is a simplified version that will be used in the mathematical model. miR-9 is upregulated in NSCLC. Although Yanaihara et al. (2006) reported a decrease of miR-9 using microarray data [6], several other papers, some more recent, reported an increase of miR-9 in NSCLC: Volinia et al. (2006) and Võsa et al. (2011) used microarray [7], [9], and Crawford et al. (2009) used PCR [8]. We have analyzed 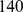 cases of NSCLC with PCR and demonstrate miR-9 overexpression in lung tumors compared to adjacent uninvolved lung and present a representation of 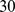 such cases; see Supplementary Material S1.

MYC controls many fundamental cellular processes, and aberrant MYC expression is known to be associated with cancer. For example, Frenzel et al. (2010) observed that MYC is usually activated in many cancers [12], and Aguda et al. (2008) showed how MYC can act as either an oncogene or tumor suppressor [13]. In lung cancer, MYC family oncogenes are amplified in both small-cell lung cancers (SCLC) and NSCLC [14], [15]. Moreover, c-MYC can induce metastasis in c-Raf mutant NSCLC [16].

Investigators have also identified a link between MYC and miRNAs that also play a significant role in cancer. Rinaldi et al. (2007) showed that both MYC and the miRNA cluster miR-17-92 are amplified in human mantle cell lymphoma [17]; Frenzel et al. (2010) described miR-9 as an oncogenic miRNA and let-7 as a tumor suppressor miRNA both of which are regulated by MYC [12]: MYC induces miR-9, which blocks tumor suppressor pathways, while MYC inhibits let-7, which blocks oncogenic pathways. Ma et al. (2010) found that miR-9 is driven by MYC, downregulates E-Cadherin, and induces metastasis in breast cancer [10]. Wolfer and Ramaswamy (2011) investigated the role of MYC in breast cancer metastasis using a signaling pathway that includes let-7, miR-9, E-Cadherin, and EMT [18].

Our proposed pathway is based on several lines of investigation. Similar to breast cancer, let-7 is downregulated in NSCLC [2], [3]. Takamizawa et al. (2004) demonstrated that reductions of let-7 as high as 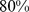 occurred in tumors compared to uninvolved adjacent lung tissue [2]. In this same study, only 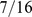 cases had such reductions (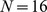). However, more recent investigation by Inamura et al. (2007) demonstrated that among well-differentiated adenocarcinomas (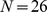), the reductions in let-7 family members were more modest (approximately 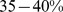) [19]. Wang et al. (2011) asserted that c-MYC represses transcription of let-7 [20]. Johnson et al. (2005) and others showed that Ras is suppressed by let-7 [21]. Lee and Dutta (2007) suggested that let-7 represses HMGA-2 in a lung cancer cell [22], and Thuault et al. (2008) asserted that HMGA-2 causes EMT by activating Snail1 which in turn represses E-Cadherin [23]. E-Cadherin downregulates MMP in bronchial tumor cells [24]. Both E-Cadherin and MMP have been implicated as biomarkers in several solid malignancies including lung cancer. A recent investigation showed that elevated levels of MMP-9 in cases of NSCLC correlated with advanced stages and the presence of metastases [25]. In addition Rao et al. (2005) demonstrated in vitro and in vivo that adenoviral mediated gene transfer of MMP-9 could reduce lung cancer invasive capacity and formation of metastases [26]. Decreased E-Cadherin expression also appears to correlate with clinically more aggressive disease [27]–[29].

Roberts and Der (2007) used an EGFR-Ras-Raf-MEK-ERK pathway to explain that 10% of NSCLC arise from EGFR mutations and that 30% of NSCLC arise from mutations in Ras [30]. SOS is an intermediate between the EGF-EGFR complex and Ras [31], and is repressed through negative feedback by ERK [32], [33]. Huang et al. (2011) showed that ERK/MAPK in lung cancer activates c-MYC [34]. Figure 1 A provides a summary of the above lines of investigation. For the purposes of simplicity, we propose a simpler version in Figure 1 B which nevertheless encompasses the main features of Figure 1 A. We recognize that other signaling pathways are driven by the EGF-EGFR complex including PI3K/Akt which regulates cell survival. However, given our interest in miR-9 and let-7 as potential biomarkers, we have not included this pathway in our model.

### Model Equations

We introduce a system of ordinary differential equations that describe a signaling pathway of EMT (represented by the level of MMP mRNA) induced by MYC through miR-9 and let-7 as shown in Figure 1 B. The differential equations 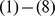 are based on Figure 1 B, and detailed explanations are given in Methods. Notation for species concentrations is given in Table 1.
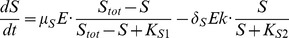
(1)

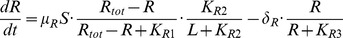
(2)

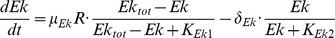
(3)

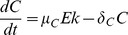
(4)

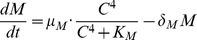
(5)

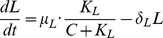
(6)

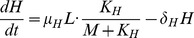
(7)

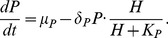
(8)


**Table 1:** Notation for species concentrations.

Notation	Description
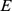	EGF-EGFR complex (constant)
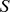	active SOS concentration
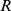	active Ras concentration
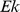	active ERK concentration
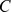	MYC protein concentration
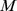	miR-9 concentration
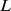	let-7 concentration
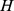	E-Cadherin concentration
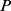	MMP mRNA concentration

The table gives notation for species concentrations that are used in the mathematical model.

### Simulations

A large number of NSCLC cases arise from EGFR mutations [35], [36] or Ras mutations [37]. We assume that negative feedback of ERK to SOS may be disrupted in NSCLC. We describe these aberrations by increasing 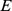, increasing 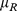, or decreasing 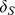, so that concentration level of EGF-EGFR complex increases, Ras is over-activated by SOS, or negative feedback of ERK to SOS is weakened. The following simulations demonstrate the effect of increase in 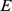 and in 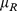 and decrease in 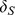 on the increase in miR-9, let-7 and MMP.

Simulations of the model equations were performed using Matlab. We used an ode solver, ode15 s, to solve a system of ordinary differential equations numerically. To solve a system of stochastic differential equations with random inputs in miR-9 or let-7 numerically, we developed a code using an Euler scheme. All initial values are taken to be those of healthy normal cells, namely, 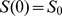, 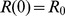, 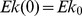, 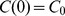, 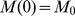, 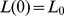, 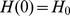, and 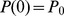.

**Figure 2:**
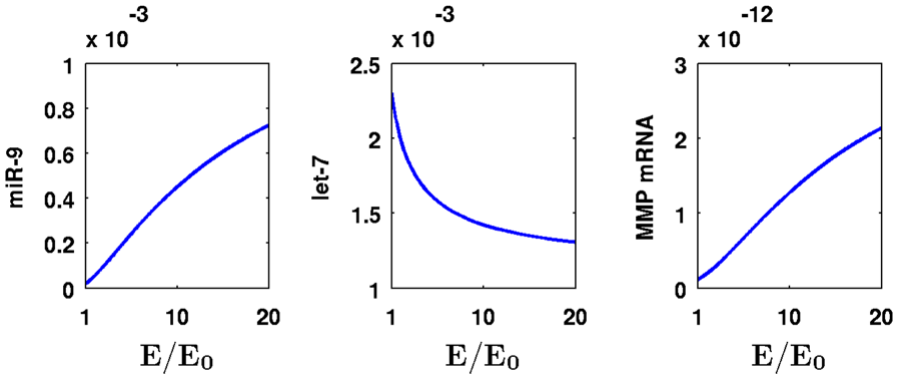
Concentration changes of miR-9, let-7, and MMP mRNA with different values for 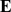
 **.** The units on the vertical axes are in 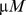 and the time is at 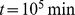.

If 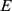 increases as a result of mutations in EGFR, we expect an increase in miR-9 and a decrease in let-7 as indeed are observed in lung cancer. There will also be an increase in MMP mRNA signifying EMT and cell migration, which contributes to metastasis. Figure 2 shows the level of miR-9, let-7, and MMP at 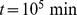 as a function of 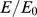: as 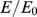 increases, miR-9 and MMP mRNA concentrations increase and let-7 concentration decreases. For example, for 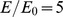, the level of miR-9 increases by 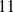-fold from 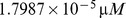 to 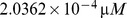 and that of MMP mRNA concentration increases by 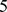-fold from 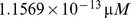 to 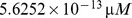 compared to the level in healthy normal cells. On the other hand, the level of let-7 concentration decreases by 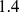-fold from 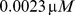 to 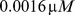.

**Figure 3:**
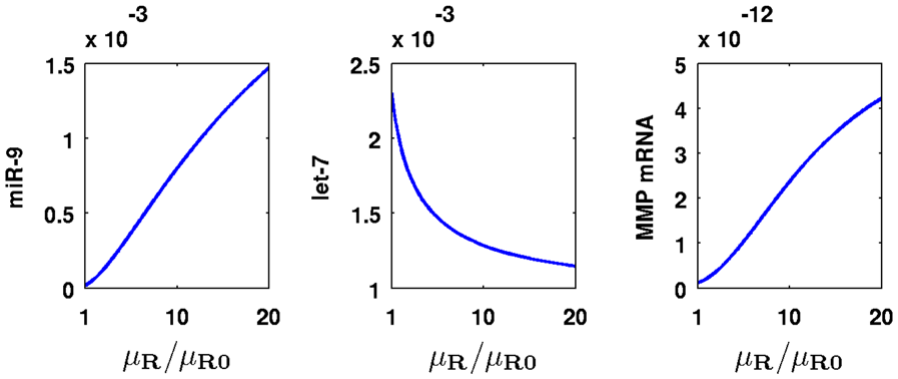
Concentration changes of miR-9, let-7, and MMP mRNA with different values for 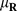
 **.** The units on the vertical axes are in 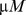 and the time is at 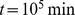.

Figure 3 shows the effect of Ras mutations on the levels of miR-9, let-7, and MMP mRNA after 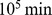. Ras mutations are represented by an increase in 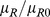. We see that as 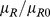 increases, so do the concentrations of miR-9 and MMP mRNA while let-7 concentration decreases. For example, for 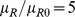, the level of miR-9 concentration increases by 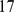-fold from 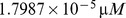 to 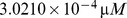 and that of MMP mRNA concentration increases by 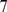-fold from 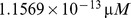 to 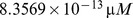 compared to the level in healthy normal cells. On the other hand, the level of let-7 concentration decreases by 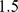-fold from 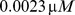 to 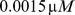.

**Figure 4:**
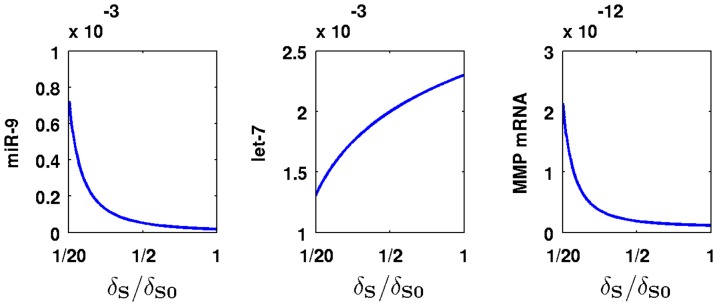
Concentration changes of miR-9, let-7, and MMP mRNA with different values for 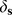
 **.** The units on the vertical axes are in 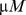 and the time is at 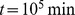.

When the negative feedback of ERK to SOS is weakened as a result of possible mutations in ERK, the parameter 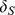 in Eq. (1) is decreased. Figure 4 shows the effect of these mutations: as 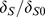 decreases, the concentrations of miR-9 and MMP increase and that of let-7 decreases. For example, for 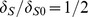, the level of miR-9 concentration increases by 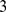-fold from 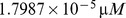 to 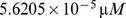 and that of MMP mRNA concentration increases by 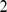-fold from 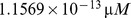 to 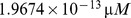 compared to the level in healthy normal cells. On the other hand, the level of let-7 concentration decreases by 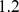-fold from 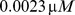 to 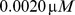.

**Figure 5:**
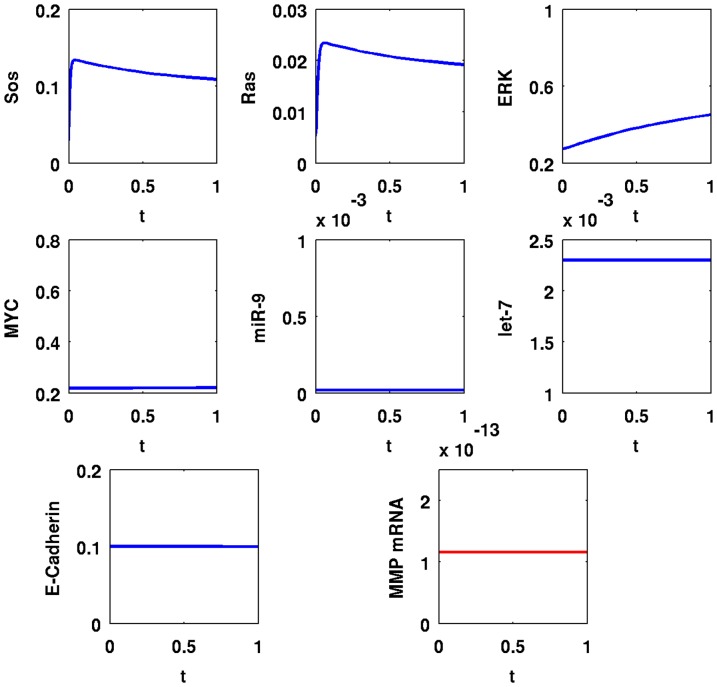
Simulation results for a cancer cell with EGFR mutations, 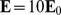
 **.** Time is from 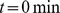 to 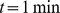; initial values are those of a normal healthy cell; the units on the vertical axes are in 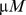 and the units on the horizontal axes are in minutes.

**Figure 6:**
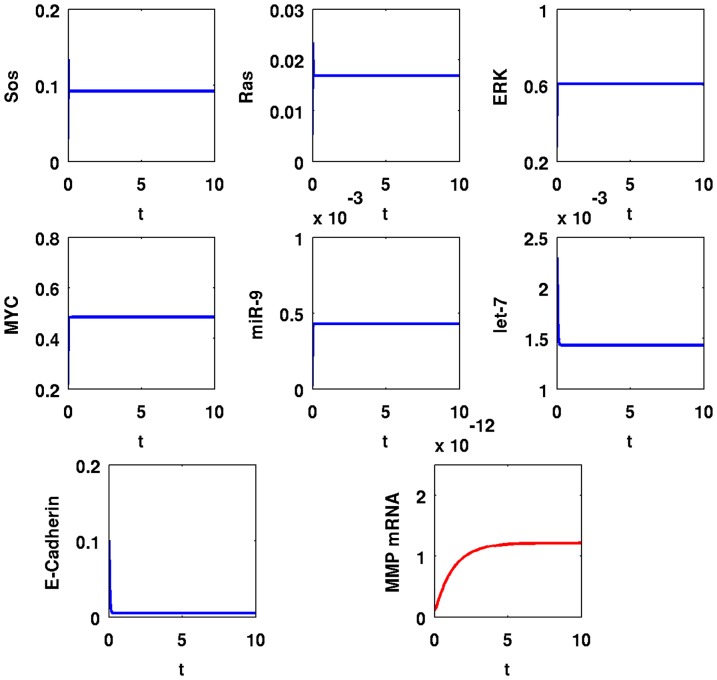
Simulation results for a cancer cell with EGFR mutations, 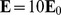
 **.** Time is from 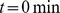 to 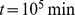; initial values are those of a normal healthy cell; the units on the vertical axes are in 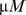 and the units on the horizontal axes are scaled in 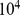 minutes.

In Figure 5, we simulate the time evolution of SOS, Ras, ERK, MYC, miR-9, let-7, E-Cadherin, and MMP mRNA over a period of 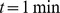 with 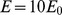; in Figure 6 the simulations are carried out for the longer period of 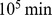. A comparison between the panels of the two figures shows that the dynamics of SOS, Ras, and ERK are very fast; MYC, miR-9, and let-7 change relatively slower, and MMP mRNA takes even longer to reach equilibrium. After 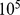 minutes, SOS and Ras increased by 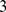-fold from 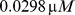 to 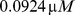 and from 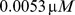 to 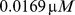, respectively; ERK and MYC increased by 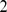-fold from 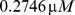 to 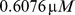 and from 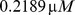 to 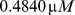, respectively; miR-9 increased by 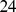-fold from 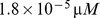 to 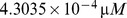; MMP increased by 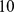-fold from 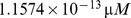 to 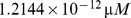 compared to their values in normal cells; let-7 decreased by 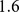-fold from 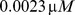 to 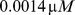, and E-Cadherin decreased by 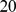-fold from 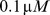 to 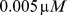.

**Figure 7:**
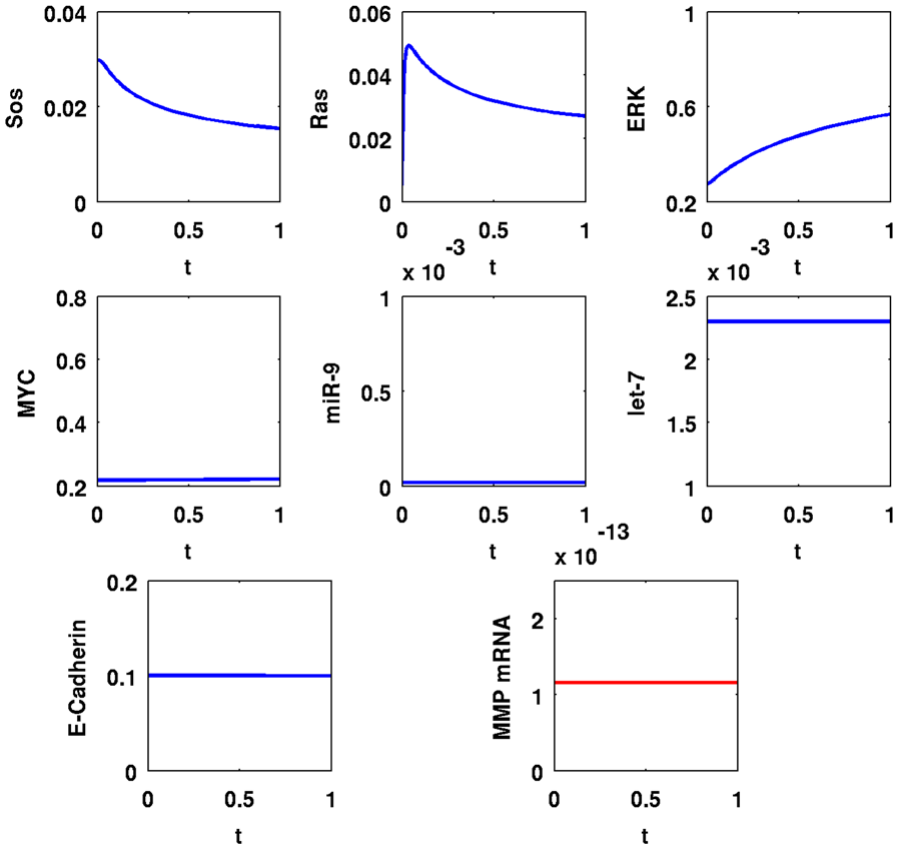
Simulation results for a cancer cell with Ras mutations, 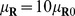
 **.** Time is from 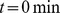 to 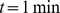; initial values are those of a normal healthy cell; the units on the vertical axes are in 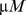 and the units on the horizontal axes are in minutes.

**Figure 8:**
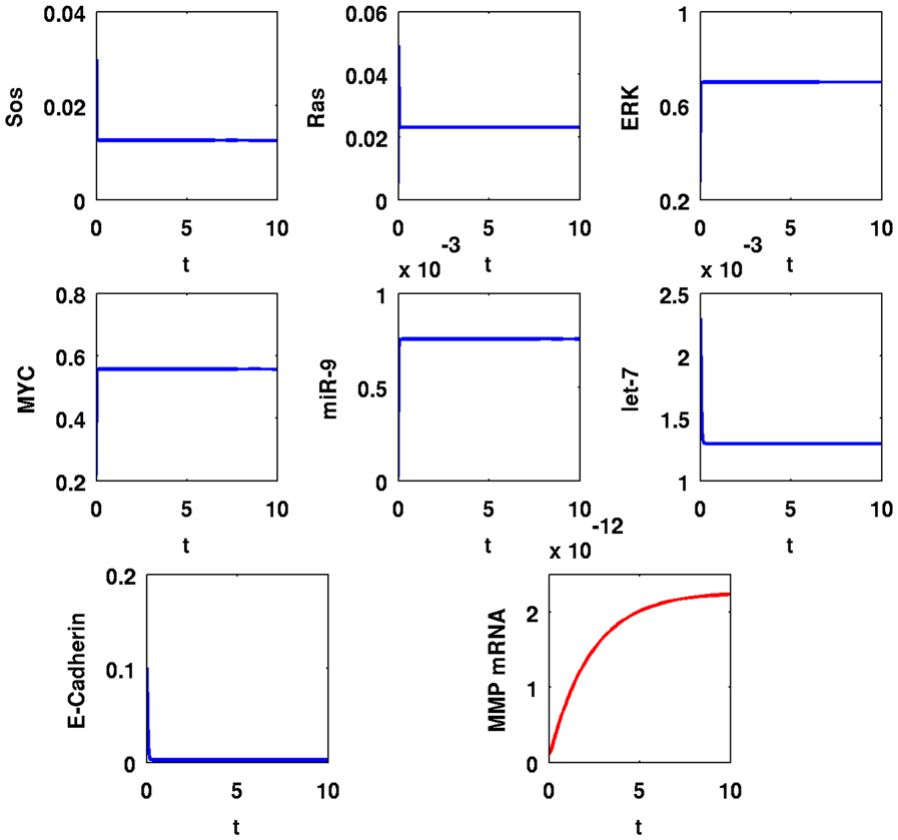
Simulation results for a cancer cell with Ras mutations, 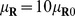
 **.** Time is from 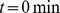 to 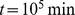; initial values are those of a normal healthy cell; the units on the vertical axes are in 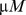 and the units on the horizontal axes are scaled in 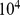 minutes.

**Figure 9:**
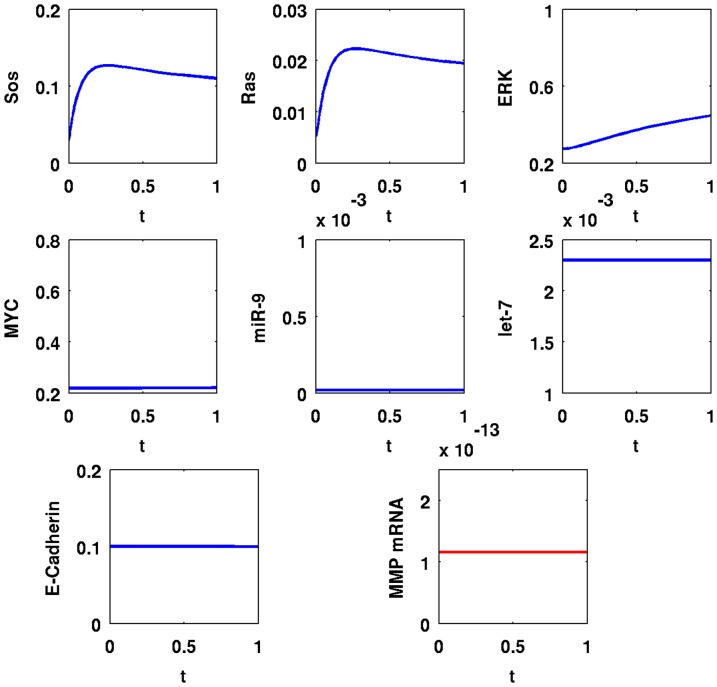
Simulation results for a cancer cell with disruption in the negative feedback from ERK to SOS, 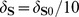
 **.** Time is from 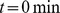 to 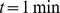; initial values are those of a normal healthy cell; the units on the vertical axes are in 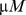 and the units on the horizontal axes are in minutes.

**Figure 10:**
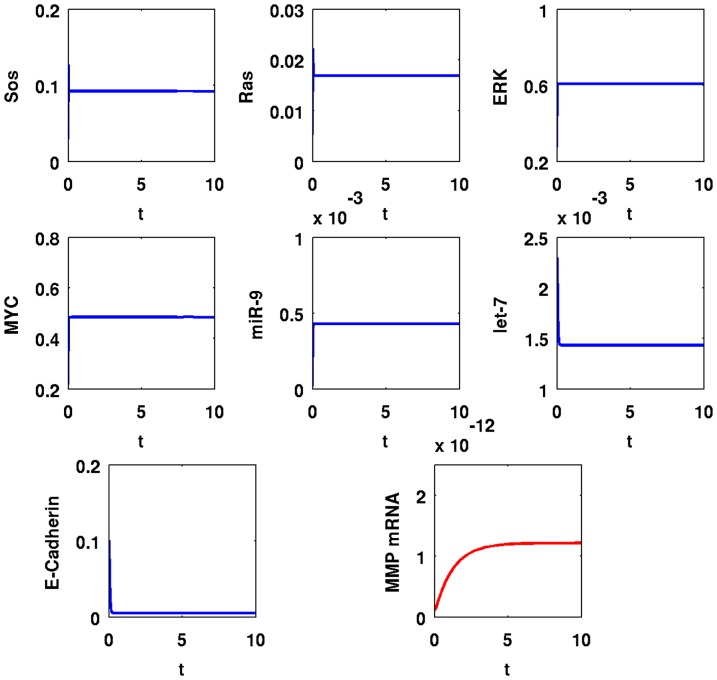
Simulation results for a cancer cell with disruption in the negative feedback from ERK to SOS, 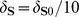
 **.** Time is from 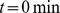 to 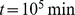; initial values are those of a normal healthy cell; the units on the vertical axes are in 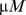 and the units on the horizontal axes are scaled in 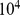 minutes.

Figures 7 and 8 show similar simulations when 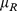 is increased to 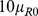 and Figures 9 and 10 show similar simulations when 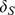 is decreased to 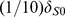. In Figure 8, Ras increased by 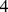-fold from 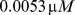 to 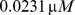; ERK and MYC increased by 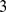-fold from 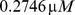 to 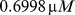 and from 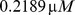 to 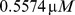, respectively; miR-9 increased by 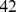-fold from 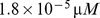 to 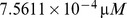; MMP increased by 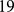-fold from 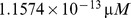 to 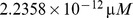 compared to their values in normal cells; SOS decreased by 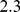-fold from 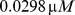 to 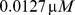; let-7 decreased by 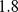-fold from 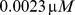 to 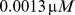, and E-Cadherin decreased by 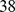-fold from 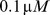 to 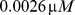. In Figure 10, concentration changes essentially in the same amount as in Figure 6.

**Figure 11:**
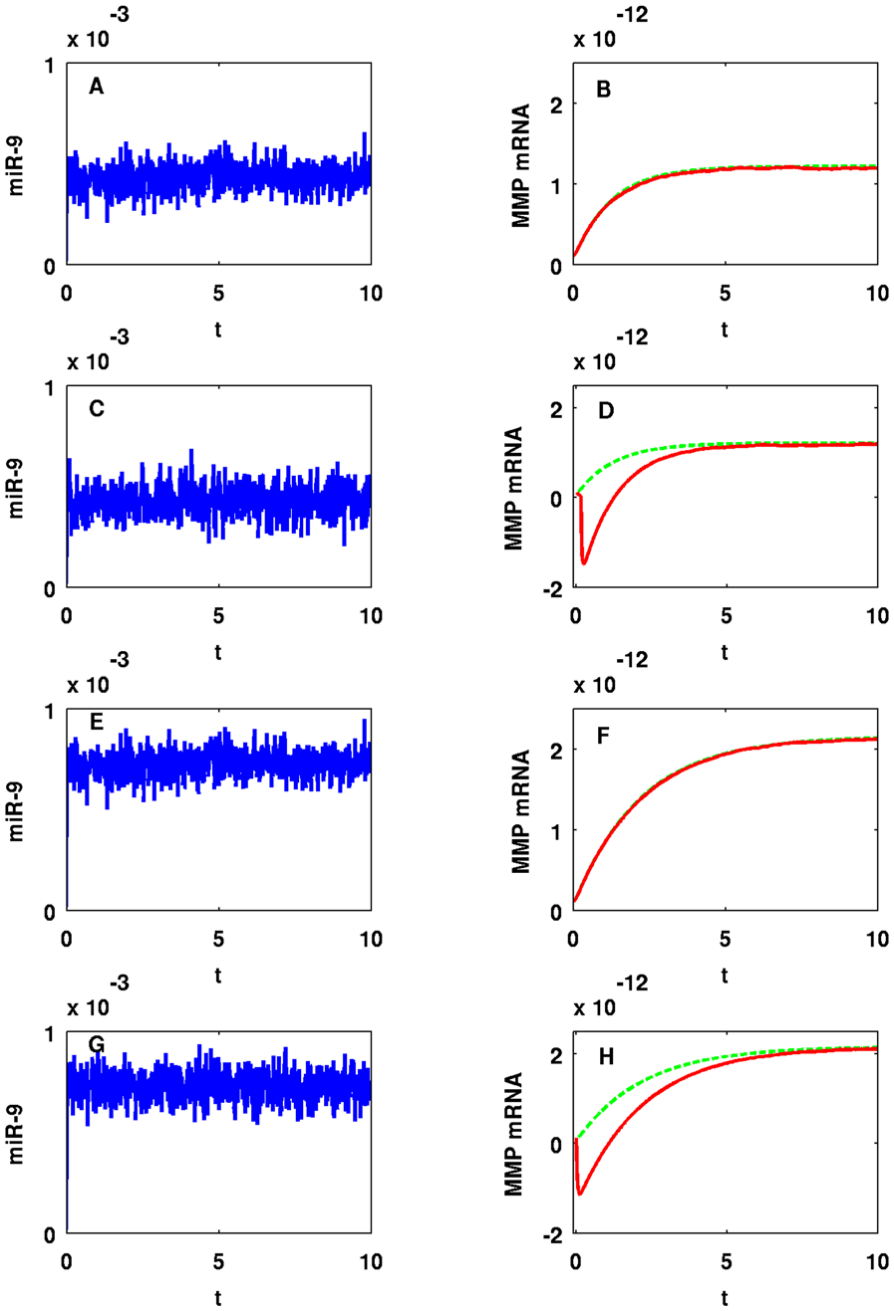
One sample path of miR-9 and MMP concentrations in time with random input in miR-9. For (A–D) 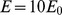 and for (E–H) 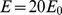. For (A, B, E, F) 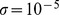 and for (C, D, G, H) 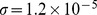. The units on the horizontal axes are scaled in 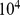 minutes.

**Figure 12:**
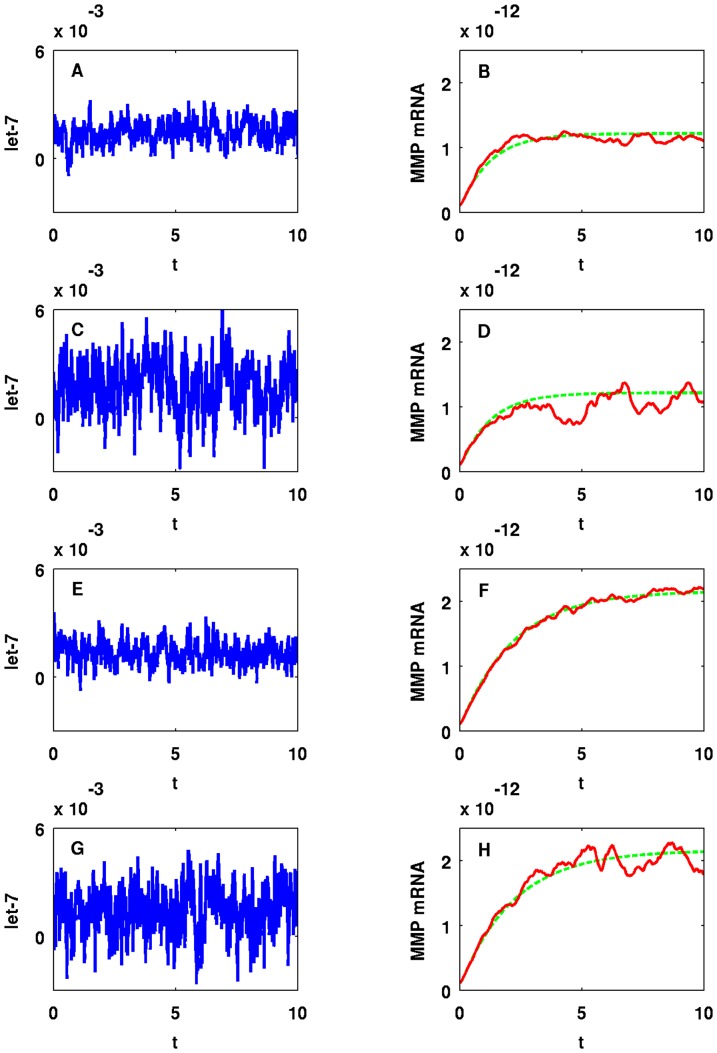
One sample path of let-7 and MMP concentrations in time with random input in let-7. For (A–D) 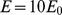 and for (E–H) 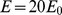. For (A, B, E, F) 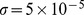 and for (C, D, G, H) 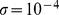. The units on the horizontal axes are scaled in 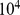 minutes.

**Figure 13:**
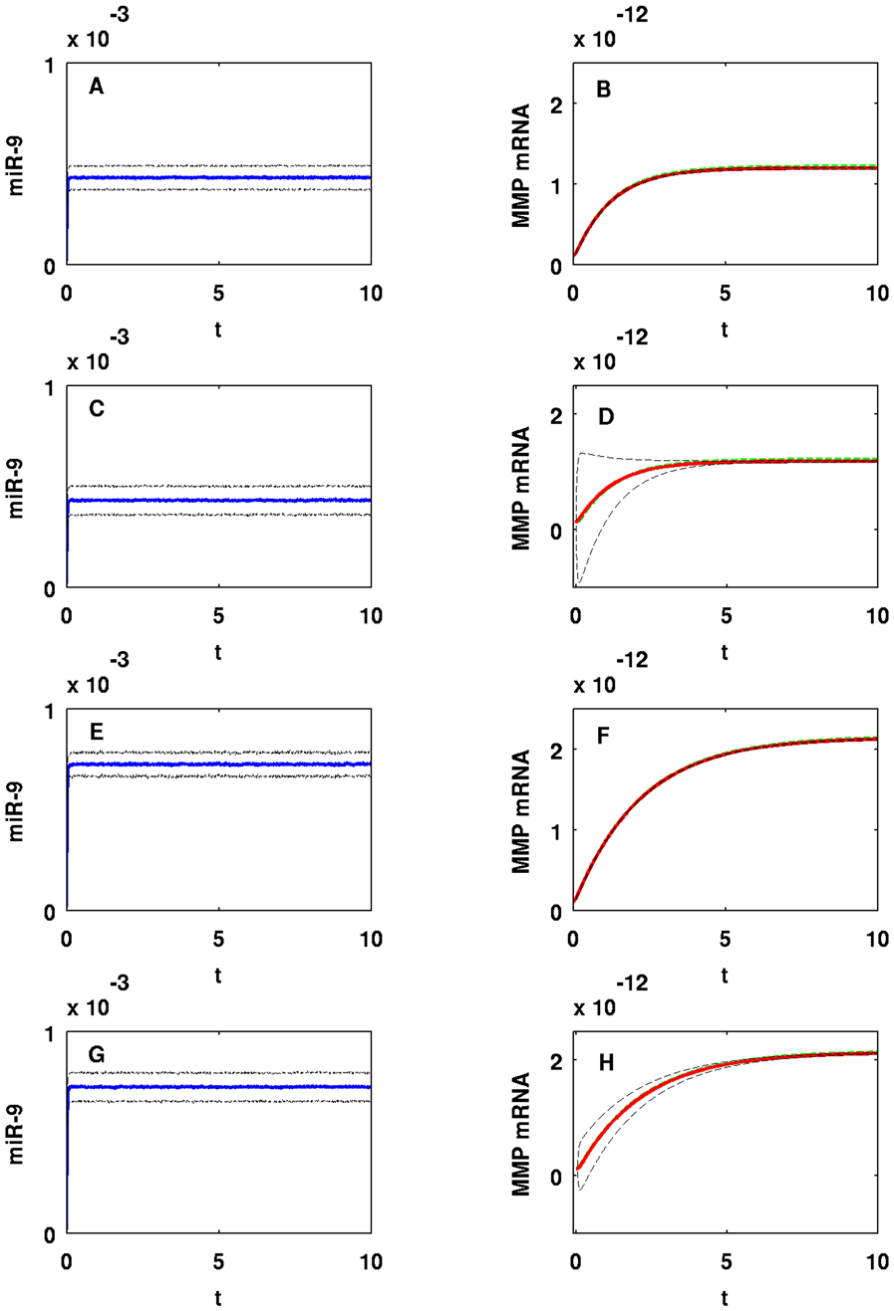
Mean concentrations of miR-9 and MMP and standard deviations from the means in time with random input in miR-9. For (A–D) 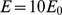 and for (E–H) 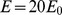. For (A, B, E, F) 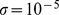 and for (C, D, G, H) 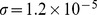. The units on the horizontal axes are scaled in 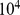 minutes. The result is taken from 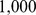 realizations of simulation.

**Figure 14:**
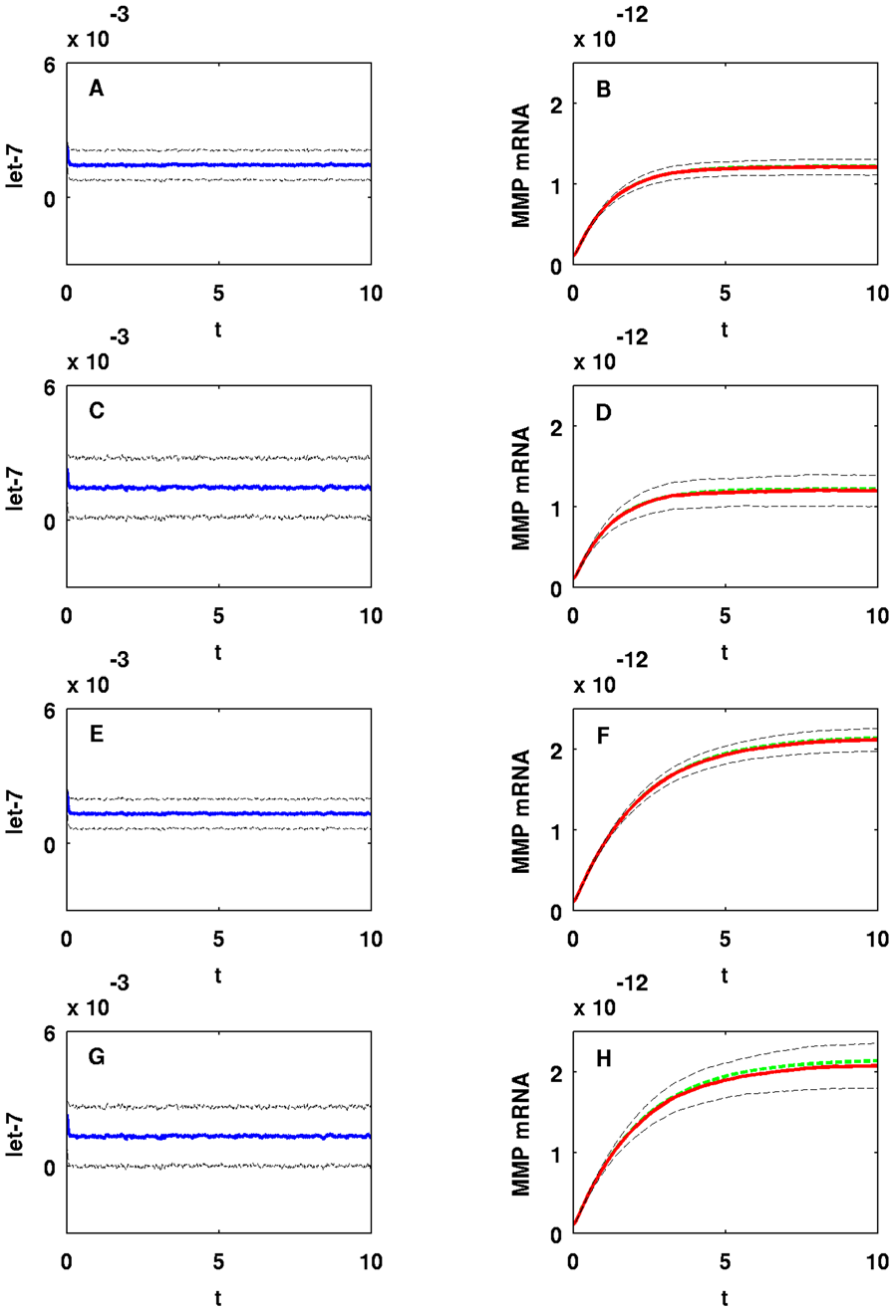
Mean concentrations of let-7 and MMP and standard deviations from the means in time with random input in let-7. For (A–D) 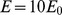 and for (E–H) 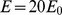. For (A, B, E, F) 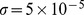 and for (C, D, G, H) 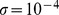. The units on the horizontal axes are scaled in 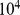 minutes. The result is taken from 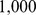 realizations of simulation.

It would be interesting to study the effect of a ‘background’ on miR-9 and let-7, namely, the genes with whom these miRNAs interact. Such interactions however, are not reported in the literature. We therefore model such interactions by a random input. Figure 11 shows how random perturbations of miR-9 affect MMP (EMT). Setting 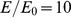 and 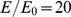 as given in Figure 2, miR-9 perturbed by random Gaussian input and MMP are shown in Figure 11 A–D and E–H, respectively (we added 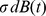 on the right-hand side of 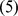 where 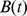 is a standard Brownian motion). Panels A/B and E/F in Figure 11 correspond to the case when miR-9 is perturbed by Gaussian input with 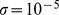 and Panels C/D and G/H in Figure 11 correspond to the case when we increase 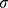 to 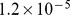. In Panels B/D/F/H in Figure 11, we compare MMP concentration with random perturbations (red line) and without perturbations (green dotted line). Figure 12 shows similar results in the case of let-7 with 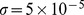 and 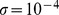. Panels A/B and E/F in Figure 12 correspond to the case when let-7 is perturbed by Gaussian input with 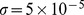 and Panels C/D and G/H in Figure 12 correspond to the case when we increase 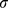 to 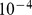. Figures 13 and 14 show means (blue or red line) and standard deviations (black dotted line) from the means of miR-9, let-7, and MMP concentrations obtained from 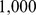 realizations of simulation with the same parameters in Figures 11 and 12. Simulation results in Figures 11-14 are obtained with fixed time step, 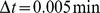.

We conclude that mean MMP concentrations and standard deviations from the means are stable (robust) to small perturbations in miR-9, i.e. when 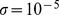. However, when we increase 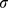 already to 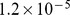 stability of standard deviations from the mean MMP concentration tends to break down as we see from Panels D/H in Figure 13; Panels D/H in Figure 11 show one sample path of unstable MMP concentration against miR-9 perturbation. On the other hand, mean MMP concentrations and standard deviations from the means are much more stable for let-7 perturbations with large 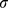, and trajectories of means closely follow the trajectory of MMP without random input as shown in Figure 14; Figure 12 shows one sample path of MMP concentration against let-7 perturbation. Notice that we have taken 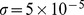 in Panels A/B/E/F and 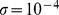 in Panels C/D/G/H. For let-7, if we take 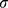 as small as 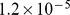 as we did in Panels C/D/G/H in Figure 11, standard deviations are very small and negligible (not shown here). The reason why MMP is more stable against random perturbations of let-7 than against miR-9 perturbations is that let-7 perturbations undergo damping by the negative feedbacks from let-7 to Ras and from ERK to SOS, as shown in Figure 1. Similar results (not shown here) hold when we vary 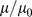 or 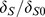, instead of 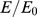.

### Sensitivity Analysis

**Figure 15:**
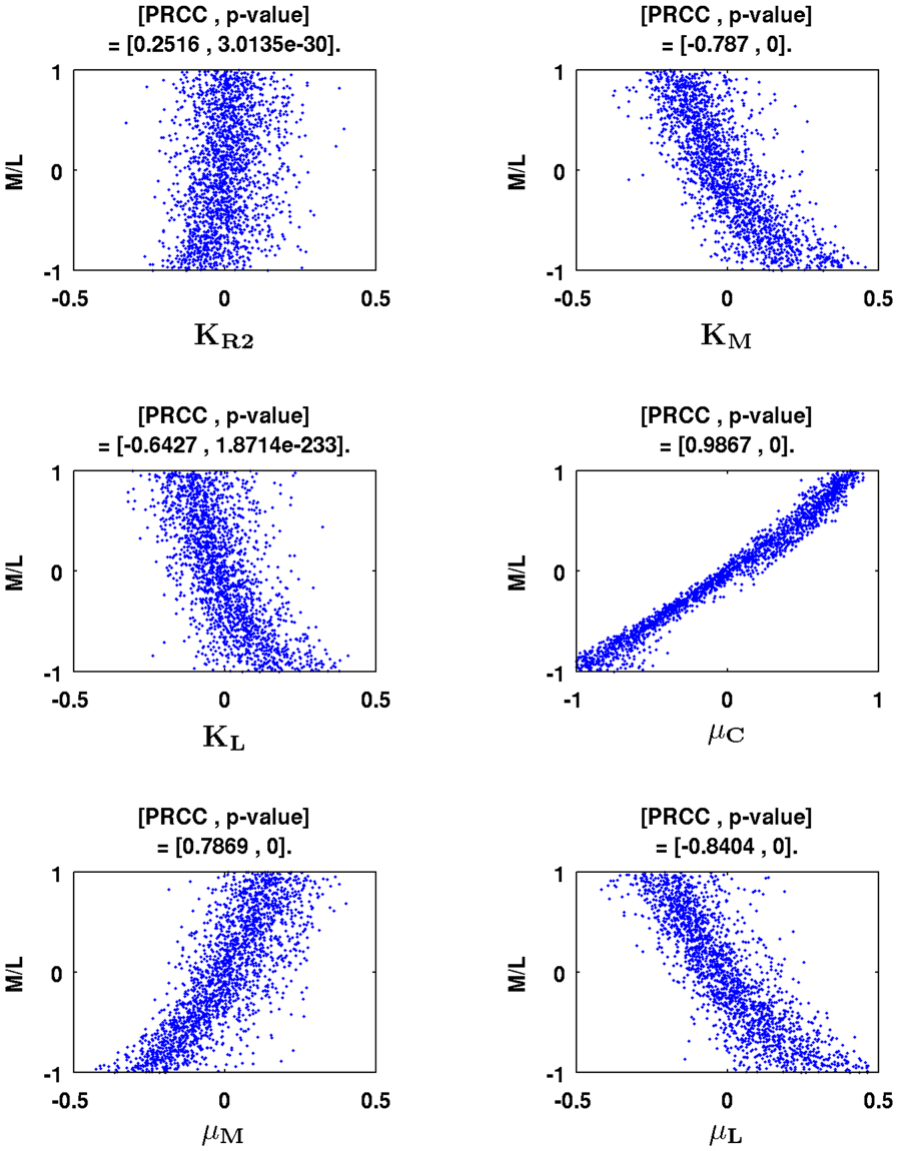
Scatter plots of rank transformed 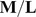 with several rank transformed parameters. Scatter plots are drawn for statistically significant parameters (p-value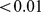); the units on the horizontal and vertical axes are scaled in 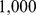; time is at 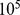 minutes and the result is taken from 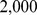 realizations of simulation.

Since we are focusing on miR-9 upregulation and let-7 downregulation as potential biomarkers for lung cancer, we wanted to determine how the quotient 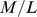 of miR-9 divided by let-7 depends on the parameters of the model equations. We focused on the 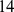 parameters in Table 2 which are only estimations. We performed sensitivity analysis, employing the method of partial rank correlation coefficient (PRCC), using previously described program [38]. We let each of the 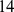 parameters vary in the interval between 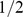 of the estimated value and twice its estimated value. Using Latin Hypercube sampling method as in [38], we sampled each parameter from uniformly distributed intervals and ran 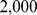 realizations of simulation. Then, we transformed the sampled parameter values and the ratio 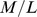 between miR-9 and let-7 as computed in the simulation to rank values, and computed partial rank correlation coefficients. PRCC values of the estimated parameters and their ranges are presented in Table 3, and scatter plots of statistically significant parameters are shown in Figure 15.

**Table 2:** Summary of the parameter values.

Name	Description	Value used	References
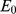	concentration of EGF-EGFR complex	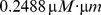	[32]
	(constant)		
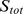	total concentration of SOS	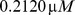	[32]
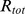	total concentration of Ras	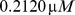	[32]
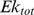	total concentration of ERK	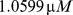	[32]
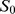	Steady-state concentration of active SOS	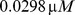	estimated
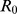	Steady-state concentration of active Ras	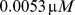	estimated
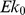	Steady-state concentration of active ERK	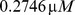	estimated
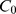	Steady-state concentration of MYC protein	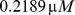	[41]
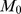	Steady-state concentration of miR-9	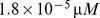	estimated
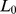	Steady-state concentration of let-7	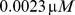	[43]
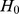	Steady-state concentration of E-Cadherin	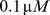	[47]
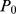	Steady-state concentration of MMP mRNA	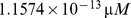	[49]
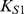	Saturation of inactive SOS on active SOS	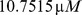	[32]
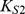	Saturation of active SOS on inactive SOS	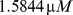	[32]
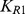	Saturation of inactive Ras on active Ras	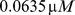	[32]
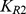	Control of let-7 on Ras	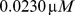	estimated
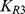	Saturation of active Ras on inactive Ras	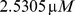	[32]
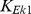	Saturation of inactive ERK on active ERK	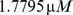	[32]
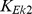	Saturation of active ERK on inactive ERK	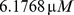	[32]
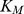	Saturation of MYC on miR-9	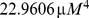	estimated
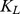	Control of MYC on let-7	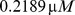	estimated
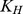	Control of MYC on E-Cadherin	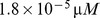	estimated
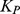	Control of E-Cadherin on MMP mRNA	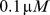	estimated
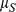	Catalytic production rate of active SOS	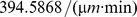	[32]
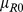	Catalytic production rate of active Ras	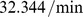	[32]
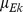	Catalytic production rate of active ERK	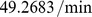	[32]
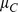	Catalytic production rate of MYC	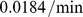	estimated
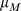	Catalytic production rate of miR-9	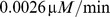	estimated
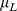	Catalytic production rate of let-7	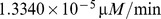	estimated
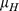	Catalytic production rate of E-Cadherin	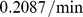	estimated
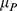	Catalytic production rate of MMP	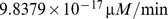	estimated
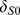	Degradation rate of active SOS	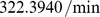	[32]
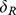	Degradation rate of active Ras	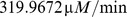	[32]
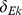	Degradation rate of active ERK	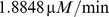	[32]
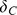	Degradation rate of MYC protein	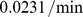	[42]
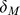	Degradation rate of miR-9	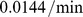	[45]
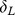	Degradation rate of let-7	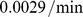	[46]
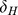	Degradation rate of E-Cadherin	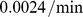	[48]
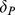	Degradation rate of MMP mRNA	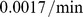	[51]

The table summarizes all the parameter values of the model equations (1)–(8).

**Table 3:** Parameter ranges and partial rank correlation coefficient (PRCC) values.

Parameter	Range	PRCC
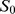	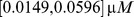	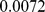
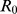	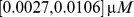	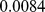
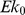	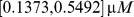	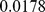
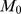	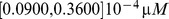	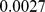
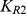	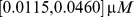	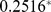
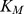	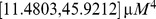	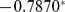
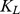	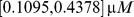	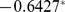
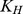	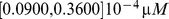	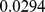
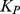	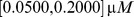	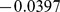
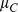	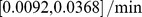	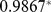
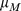	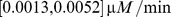	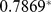
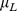	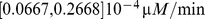	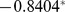
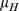	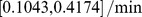	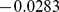
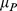	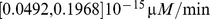	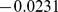

Statistically significant parameters are denoted as ^*^ (p-value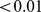).

Among the 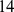 parameters, 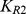, 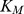, 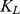, 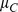, 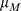, and 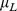 were statistically significant. The parameters 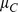 and 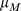 were strongly positively correlated with 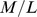. This is natural; indeed 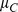 and 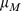 are production rates of MYC and miR-9. As we increase production rate of MYC, miR-9 concentration increases and let-7 concentration decreases. On the other hand, 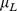, 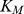, and 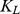 were strongly negatively correlated to 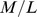. This is also to be expected. Indeed, 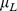 is the production rate of let-7, 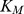 is the saturation constant of MYC as source for miR-9, and 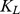 is the control constant of MYC in the let-7 equation. Therefore, it is natural that 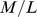 would decrease as the parameters 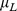, 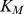, and 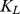 increase. When we ran 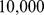 realizations of simulation, we obtained similar results.

### EGFR inhibition reduces both c-MYC and miR-9 in a concentration dependent manner

**Figure 16:**
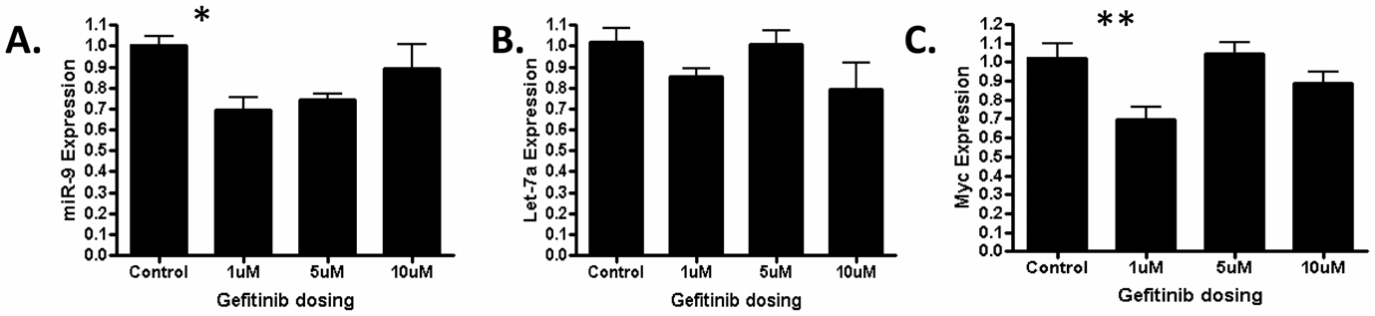
QRT-PCR expression of miR-9, let-7a and c-MYC in HCC827 lung cancer cell lines treated with Gefitinib. Statistical significance is defined as *p<0.05 in (A) and **p<0.01 in (C).

In an initial attempt to validate our mathematical model, we treated an EGFR mutant lung cancer cell line with several concentration of the clinically used EGFR inhibitor Gefitinib. We then assessed treated cells for miR-9, let-7a and c-MYC expression by QRT-PCR. As shown in Figure 16, we determined that while lower concentrations (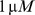) of Gefitinib caused a statistically significant reduction in both miR-9 and c-MYC, similar effects were not evident at higher concentrations of Gefitinib or in let-7a. These findings while they would need to be validated in other cell lines suggest the additional complexity of the effects EGFR inhibition on miRNA expression and that our mathematical model only partially predicts the biological links between EGFR, c-MYC and miRNA in lung cancer.

## Discussion

Lung cancer is the leading cause of cancer-related deaths worldwide. The majority of cases are diagnosed at later stages thus limiting therapeutic options and contributing to poor outcome. As a result, investigators have sought to identify lung cancer specific biomarkers that may be utilized for early detection and to better understand the metastatic process. Such biomarkers may significantly improve prognosis and reduce mortality. In this paper, we have proposed a mathematical model that integrates the miRNAs let-7 and miR-9 into the process of EMT. miR-9 has been shown to be significantly upregulated and let-7 downregulated in NSCLC.

Based on the experimental literature, we introduced a signaling pathway from the EGF-EGFR complex to MMP expression which involves SOS, Ras, ERK, MYC, the miRNAs miR-9 and let-7, E-Cadherin, and MMP. Recent studies have demonstrated elevated MMP-9 in NSCLC [25], but for modeling purposes we have referred to MMP in a generic manner. Using an EGFR mutant lung cancer cell line, we showed that inhibition of EGFR leads to a reduction in miR-9 as well as c-MYC expression. However, the relationships between miR-9 and c-MYC were not consistent at higher concentrations of drug treatment. These findings support the complexity of the kinetics of miRNA and target gene relationships and highlight the inherent difficulties with modeling miRNA biology. Our findings suggest that higher concentrations of EGFR are likely to engage other regulators of miR-9 and/or c-MYC and that miR-9 may be under the regulatory control of additional genes beyond c-MYC.

We correspondingly developed a mathematical model including a system of differential equations and used the model to compute the level of miR-9 overexpression and let-7 downexpression in the setting of EGFR mutations and Ras mutations. We showed that such mutations upregulate the level of miR-9 and downregulate the level of let-7. The 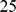-fold increase in miR-9 levels obtained in the simulations was consistent quantitatively with clinical data reported in human lung tumors (Supplementary Material S1). Our experiments with EGFR mutant lung cancer cells did not show any significant changes in let-7 suggesting that let-7 may also be regulated by other signaling networks. We investigated how random perturbations of let-7 and miR-9 affect MMP and concluded that MMP is more robust against let-7 perturbations than against miR-9 perturbations; this can be explained by the fact that let-7 perturbations undergo damping by the negative feedbacks from let-7 to Ras and from ERK to SOS.

To the best of our knowledge, the present paper is the first one that develops a model for lung cancer and miRNA in terms of differential equations. The model is based on a signaling pathway that includes miR-9 and let-7. Simulations of the model demonstrate how mutations that are detected in NSCLC include upregulation of miR-9 and downregulation of let-7. The mathematical model could be further extended by including additional signaling pathways, specifically involving let-7, that are associated with lung cancer. However, an important next step in this line of investigation is to determine how deregulation of miR-9 and let-7 may jointly contribute to lung cancer progression and may be used as reliable biomarkers. In order to address this challenge mathematically, additional clinical investigation will be required.

## Methods

In this model, we assume that the EGF-EGFR complex is at steady state and set it as a constant. Brown et al. (2004) modeled EGFR signaling with negative feedback of ERK to SOS [32]. We simplified some parts of their model to obtain the equations for SOS, Ras, and ERK. We denote by 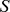, 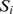, and 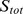 the concentrations of active SOS, inactive SOS, and total SOS, respectively. Assuming that the total number of SOS is conserved, we have
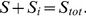
(9)


We denote by 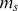 the activation rate of the inactive SOS and by 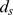 as the deactivation rate of the active SOS. Describing these conversions by the Michaelis-Menten kinetics, the governing equation for the concentration of the active SOS is given by
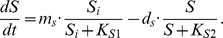



Using the fact that the EGF-EGFR complex activates SOS and that ERK represses active SOS, we replace 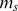 by 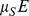 and 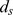 by 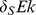, and we get Eq. (1). Similarly, we describe conversions between active and inactive Ras and between active and inactive ERK using Michaelis-Menten kinetics, and derive Eqs. (2) and (3). Here, catalytic activation rates of Ras and ERK are proportional to active SOS and active Ras concentrations, respectively. In Eq. (2), repression by let-7 of the activation of Ras is described by an inhibition factor, 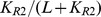. In Eq. (4), production of MYC is proportional to active ERK concentration. In Eq. (5), activation of miR-9 by MYC is described by the fourth-order Hill function, since MYC is a transcription factor and miR-9 activation may involve several enzymatic steps. In Eq. (6), let-7 production is inhibited by MYC. In Eq. (7), E-Cadherin production is proportional to let-7 concentration and is inhibited by miR-9. Throughout Eqs. (4)–(7), degradation of species is described by linear mass action kinetics. Finally, in Eq. (8) MMP is produced at constant rate and is degraded by E-Cadherin.

The parameters of Eqs. (1)–(8) are derived in the following subsections. Most of the parameters are taken from Brown et al. (2004) [32]. In their model, they have taken the initial concentrations of all active signaling species to be zero, and the initial concentrations of all inactive signaling species to be 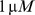 except for MEK and ERK, whose concentrations were taken to be 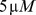. As for the EGF-EGFR complex concentration, Brown et al (2004) [32] assume it to be a variable but in our model, it is constant. This constant is chosen as the steady state concentration of the EGF-EGFR complex computed using their parameters.

### Computation of 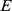


We denote by 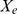, 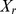, and 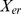 the numbers of molecules of EGF, free EGFR, and EGF-EGFR complex, and by 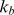 and 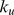 the binding and unbinding rates for the EGF-EGFR complex. If 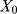 is the total number of the EGFR molecules, then 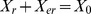. Assuming that binding and unbinding of EGF and EGFR are balanced at steady state, we have
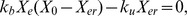
which gives
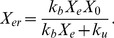
(10)


According to Brown et al. (2004) [32],
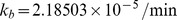


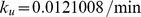


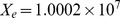


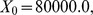
and hence 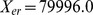. We shall determine 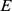 by converting 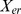 into a unit of concentration. Lung cells size, however, vary up to 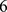-fold differences [39]. We therefore use an “average” cell size by taking it to be the HeLa cell.

Since EGF and EGFR are located on the cell surface, we need to compute the cell surface area; we assume that the cells have spherical shape with radius 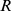. For HeLa cell, the total volume is
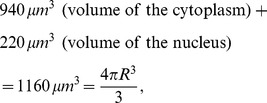
according to Fujioka et al. (2006) [40]. Hence 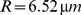 and its surface area is
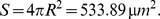



Converting the number of molecules of 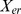 into concentration on the cell surface, we compute steady-state concentration of EGF-EGFR complex as
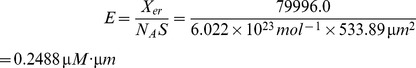
where 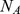 is the Avogadro's number, 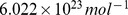; 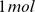 is the amount of a substance that contains as many entities as there are atoms in 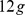 of 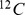, and 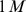 is 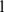 molar concentration (per liter),
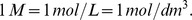



### Other parameters in the SOS equation

Let 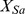 and 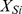 denote the numbers of active and inactive SOS molecules. According to Brown et al. (2004),
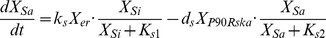
(11)where P90Rsk is a p90 ribosomal s6 kinase that inactivates SOS, and 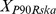 is the number of active P90Rsk molecules [32]. In that paper, parameters are given as 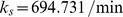, 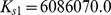, 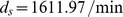, and 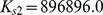. Using these numbers, we determine our parameters by
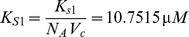


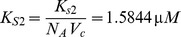


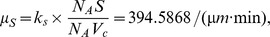
where 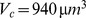 is the volume of the cytoplasm in a HeLa cell. The total number of molecules of active P90Rsk was taken to be 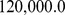 [32]. Since the initial concentration of ERK, 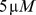, corresponds to 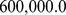 molecules, we get
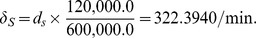



The initial concentration of SOS (all inactive) was 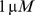, which corresponds to 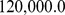 molecules. We convert this number to concentration using the volume of the cytoplasm in a HeLa cell,
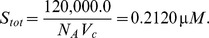



### Parameters in the Ras equation

Let 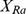 and 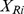 denote the numbers of molecules of active and inactive Ras. From Brown et al. (2004),
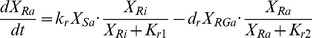
(12)where 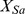 and 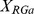 denote the numbers of molecules of active SOS and active Ras-Gap [32]. In [32], parameters are given as 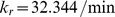, 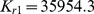, 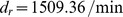, and 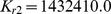. Also, the number of molecules of active Ras-Gap is treated as a constant equal to 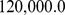. Accordingly, we determine our parameters by
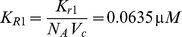


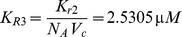


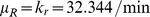


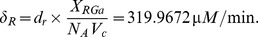



For total Ras concentration, we convert the total number of Ras molecules in a cell obtained from [32] to concentration using the volume of the cytoplasm in a HeLa cell,
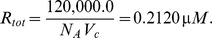



### Parameters in the ERK equation

Let 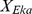 and 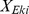 denote the numbers of molecules of active and inactive ERK. Following Brown et al. (2004),
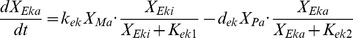
(13)where 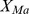 and 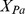 denote the numbers of molecules of active MEK and active PP2A [32]. PP2A is protein phosphatase 2 which is an enzyme targeting proteins in oncogenic signaling pathways. In that paper, parameters are given as 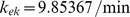, 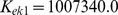, 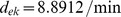, and 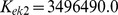; the initial total numbers of molecules of MEK and Ras are given as 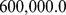 and 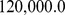, and the number of molecules of active PP2A is treated as a constant equal to 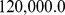. Therefore, we determine our parameters by
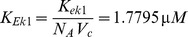


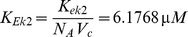


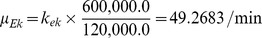


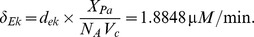



We convert the total number of ERK molecules, consisting of active and inactive ERK in a cell to concentration, using the volume of the cytoplasm in a HeLa cell, and set
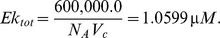



### Parameters in the MYC equation

Following Rudolph et al. (1999), there are 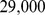 c-MYC proteins in the nucleus [41]. We convert this to concentration using the volume of the nucleus in a HeLa cell, 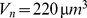. Treating this concentration as the steady-state concentration of MYC, we get
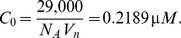



Half-life of c-MYC protein is 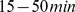 [42]. We take the half-life of c-MYC as 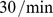, and compute a degradation rate as
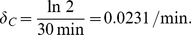



In steady state in Eq. (4),
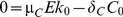
(14)where 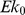 is the steady-state concentration of ERK. To determine 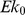, we first compute steady-state concentration of let-7. Following Lim et al. (2003), there are 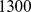 let-7 molecules in a human HeLa cell [43], and we assume that this number is at steady state. We convert it to concentration by
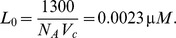



We compute a solution of Eq. (1)–(3) for 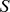, 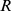, and 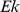 with 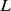 replaced by 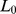 using Matlab, and obtain the steady-state concentration of ERK as 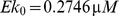. From Eq. (14), we then get
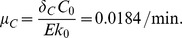



### Parameters in the miR-9 equation

Since the miR-9 copy number in the normal lung cell is very small [44], we take the steady-state concentration of miR-9 to be 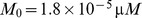. Half-life of miR-9 in human brain tissue is 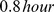 [45], which gives the degradation rate 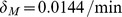. In steady state in Eq. (5),
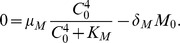



Based on the fact that miR-9 expression in the NSCLC tissues is about 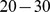 times that of normal tissues (see Supplementary Material S1), we take 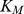 to be very large, namely, 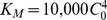. Then
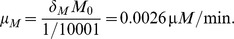



### Parameters in the let-7 equation

Half-life of let-7 after TAM treatment is 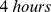 [46]. Accordingly, we take the degradation rate of 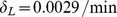. Then, in steady state in Eq. (6),
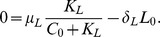



Taking 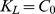 gives
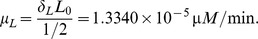



### Parameters in the E-Cadherin equation

Using the total E-Cadherin concentration in Chaplain (2011) [47], we set steady-state concentration, 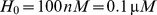. Half-life of E-Cadherin is 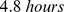 [48], so the degradation rate is 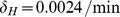. In steady state in Eq. (7),
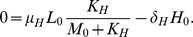



Taking 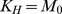 gives
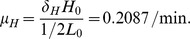



### Parameters in the MMP mRNA equation

According to Safranek et al. (2009), the number of MMP-9 mRNA in human lung tissue is 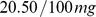 [49]. Using the human lung tissue density of 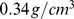 [50], we compute the MMP mRNA concentration in steady state,
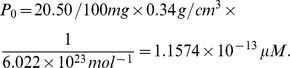



Half-life of MMP-9 mRNA is 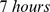 [51], so the degradation rate is 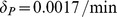. Using the steady state equation for MMP concentration,
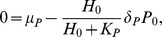
and taking 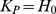, we get
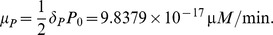



### Cell culture and drug treatment

For our experiments shown in Figure 16, we obtained the EGFR mutant lung cancer cell line (HCC827) (E746-A750 deletion) as a generous gift from our collaborator (Michela Garofalo, OSU). Cells were maintained in appropriate media. HCC827 cell lines were subsequently treated with Gefitinib (generous gift from Michela Garofalo, OSU) at concentrations of 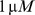, 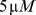, and 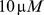. Following 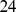 hours of exposure, cells were harvested for RNA and assessed for miR-9 (Assay ID# Hs000583), let-7a (Assay ID# Hs00377), c-MYC (Assay ID# Hs00153408_m1) (Applied Biosystems) by QRT-PCR. For miRNA assessment RNU48 was used as the endogenous control and for c-MYC GAPDH was used. Data is presented as fold difference based on 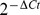. Statistical analyses were performed using ANOVA with Tukey Post Hoc analysis.

## Supporting Information

Material S1 **Experimental results of miR-9 in lung tumor tissues.** Experimental results using quantitative reverse transcription polymerase chain reaction and in situ hybridization for miR-9 are provided.
(PDF)

## References

[1] Siegel R, Naishadham D, Jemal A (2012) Cancer statistics, 2012. CA: A Cancer Journal for Clinicians. 10.3322/caac.20138 22237781

[2] Takamizawa J, Konishi H, Yanagisawa K, Tomida S, Osada H, et al (2004) Reduced expression of the let-7 microRNAs in human lung cancers in association with shortened postoperative survival. Cancer Research 64: 3753. 15172979 10.1158/0008-5472.CAN-04-0637

[3] Nicoloso M, Spizzo R, Shimizu M, Rossi S, Calin G (2009) MicroRNAs-the micro steering wheel of tumour metastases. Nature Reviews Cancer 9: 293–302. 19262572 10.1038/nrc2619

[4] Trang P, Medina P, Wiggins J, Ruffino L, Kelnar K, et al (2009) Regression of murine lung tumors by the let-7 microRNA. Oncogene 29: 1580–1587. 19966857 10.1038/onc.2009.445 PMC2841713

[5] Trang P, Wiggins J, Daige C, Cho C, Omotola M, et al (2011) Systemic delivery of tumor suppressor microrna mimics using a neutral lipid emulsion inhibits lung tumors in mice. Molecular Therapy 19: 1116–1122. 21427705 10.1038/mt.2011.48 PMC3129804

[6] Yanaihara N, Caplen N, Bowman E, Seike M, Kumamoto K, et al (2006) Unique microRNA molecular profiles in lung cancer diagnosis and prognosis. Cancer Cell 9: 189–198. 16530703 10.1016/j.ccr.2006.01.025

[7] Volinia S, Calin G, Liu C, Ambs S, Cimmino A, et al (2006) A microRNA expression signature of human solid tumors defines cancer gene targets. Proceedings of the National Academy of Sciences of the United States of America 103: 2257. 16461460 10.1073/pnas.0510565103 PMC1413718

[8] Crawford M, Batte K, Yu L, Wu X, Nuovo G, et al (2009) MicroRNA 133B targets pro-survival molecules MCL-1 and BCL2L2 in lung cancer. Biochemical and Biophysical Research Communications 388: 483–489. 19654003 10.1016/j.bbrc.2009.07.143 PMC2824514

[9] Võsa U, Vooder T, Kolde R, Fischer K, Välk K, et al (2011) Identification of miR-374a as a prognostic marker for survival in patients with early-stage nonsmall cell lung cancer. Genes, Chromosomes and Cancer 10.1002/gcc.20902 21748820

[10] Ma L, Young J, Prabhala H, Pan E, Mestdagh P, et al (2010) miR-9, a MYC/MYCN-activated microRNA, regulates E-cadherin and cancer metastasis. Nature Cell Biology 12: 247–256. 20173740 10.1038/ncb2024 PMC2845545

[11] Mascaux C, Laes J, Anthoine G, Haller A, Ninane V, et al (2009) Evolution of microRNA expression during human bronchial squamous carcinogenesis. European Respiratory Journal 33: 352. 19010987 10.1183/09031936.00084108

[12] Frenzel A, Lovén J, Henriksson M (2010) Targeting MYC-regulated miRNAs to combat cancer. Genes & Cancer 1: 660. 21779463 10.1177/1947601910377488 PMC3092216

[13] Aguda B, Kim Y, Piper-Hunter M, Friedman A, Marsh C (2008) MicroRNA regulation of a cancer network: consequences of the feedback loops involving miR-17-92, E2F, and Myc. Proceedings of the National Academy of Sciences of the United States of America 105: 19678. 19066217 10.1073/pnas.0811166106 PMC2598727

[14] Yokota J, Wada M, Yoshida T, Noguchi M, Terasaki T, et al (1988) Heterogeneity of lung cancer cells with respect to the amplification and rearrangement of myc family oncogenes. Oncogene 2: 607. 2838790

[15] Broers J, Viallet J, Jensen S, Pass H, Travis W, et al (1993) Expression of c-myc in progenitor cells of the bronchopulmonary epithelium and in a large number of non-small cell lung cancers. American Journal of Respiratory Cell and Molecular Biology 9: 33. 8393325 10.1165/ajrcmb/9.1.33

[16] Rapp U, Korn C, Ceteci F, Karreman C, Luetkenhaus K, et al (2009) MYC is a metastasis gene for non-small-cell lung cancer. PLoS One 4: e6029. 19551151 10.1371/journal.pone.0006029 PMC2696940

[17] Rinaldi A, Poretti G, Kwee I, Zucca E, Catapano C, et al (2007) Concomitant MYC and microRNA cluster miR-17-92 (C13orf25) amplification in human mantle cell lymphoma. Leukemia & Lymphoma 48: 410. 17325905 10.1080/10428190601059738

[18] Wolfer A, Ramaswamy S (2011) MYC and metastasis. Cancer Research 71: 2034. 21406394 10.1158/0008-5472.CAN-10-3776 PMC3089000

[19] Inamura K, Togashi Y, Nomura K, Ninomiya H, Hiramatsu M, et al (2007) let-7 microRNA expression is reduced in bronchioloalveolar carcinoma, a non-invasive carcinoma, and is not correlated with prognosis. Lung Cancer 58: 392–396. 17728006 10.1016/j.lungcan.2007.07.013

[20] Wang Z, Lin S, Li J, Xu Z, Yao H, et al (2011) MYC protein inhibits transcription of the microRNA cluster MC-let-7a-1 – let-7d via noncanonical e-box. Journal of Biological Chemistry 286: 39703–39714. 21903590 10.1074/jbc.M111.293126 PMC3220549

[21] Johnson S, Grosshans H, Shingara J, Byrom M, Jarvis R, et al (2005) RAS is regulated by the let-7 microRNA family. Cell 120: 635–647. 15766527 10.1016/j.cell.2005.01.014

[22] Lee Y, Dutta A (2007) The tumor suppressor microRNA let-7 represses the HMGA2 oncogene. Genes & Development 21: 1025–1030. 17437991 10.1101/gad.1540407 PMC1855228

[23] Thuault S, Tan E, Peinado H, Cano A, Heldin C, et al (2008) HMGA2 and Smads co-regulate SNAIL1 expression during induction of epithelial-to-mesenchymal transition. Journal of Biological Chemistry 283: 33437. 18832382 10.1074/jbc.M802016200 PMC2662269

[24] Nawrocki-Raby B, Gilles C, Polette M, Bruyneel E, Laronze J, et al (2003) Upregulation of MMPs by soluble E-cadherin in human lung tumor cells. International Journal of Cancer 105: 790–795. 12767064 10.1002/ijc.11168

[25] Zheng S, Chang Y, Hodges K, Sun Y, Ma X, et al (2010) Expression of KISS1 and MMP-9 in non-small cell lung cancer and their relations to metastasis and survival. Anticancer Research 30: 713–718. 20392988

[26] Rao J, Gondi C, Chetty C, Chittivelu S, Joseph P, et al (2005) Inhibition of invasion, angiogenesis, tumor growth, and metastasis by adenovirus-mediated transfer of antisense uPAR and MMP-9 in non–small cell lung cancer cells. Molecular Cancer Therapeutics 4: 1399. 16170032 10.1158/1535-7163.MCT-05-0082 PMC1343495

[27] Sulzer M, Leers M, van NOORD J, Bollen E, Theunissen P (1998) Reduced Ecadherin expression is associated with increased lymph node metastasis and unfa29 vorable prognosis in non-small cell lung cancer. American Journal of Respiratory and Critical Care Medicine 157: 1319–1323. 9563756 10.1164/ajrccm.157.4.9703099

[28] Mitselou A, Batistatou A, Nakanishi Y, Hirohashi S, Vougiouklakis T, et al (2010) Comparison of the dysadherin and E-cadherin expression in primary lung cancer and metastatic sites. Histology and Histopathology 25: 1257–1267. 20712010 10.14670/HH-25.1257

[29] Saad A, Yeap B, Thunnissen F, Pinkus G, Pinkus J, et al (2008) Immunohistochemical markers associated with brain metastases in patients with nonsmall cell lung carcinoma. Cancer 113: 2129–2138. 18720359 10.1002/cncr.23826 PMC2597625

[30] Roberts P, Der C (2007) Targeting the Raf-MEK-ERK mitogen-activated protein kinase cascade for the treatment of cancer. Oncogene 26: 3291–3310. 17496923 10.1038/sj.onc.1210422

[31] Zhao C, Du G, Skowronek K, Frohman M, Bar-Sagi D (2007) Phospholipase D2-generated phosphatidic acid couples EGFR stimulation to Ras activation by Sos. Nature Cell Biology 9: 707–712. 10.1038/ncb1594 17486115

[32] Brown K, Hill C, Calero G, Myers C, Lee K, et al (2004) The statistical mechanics of complex signaling networks: nerve growth factor signaling. Physical Biology 1: 184. 16204838 10.1088/1478-3967/1/3/006

[33] Orton R, Adriaens M, Gormand A, Sturm O, Kolch W, et al (2009) Computational modelling of cancerous mutations in the EGFR/ERK signalling pathway. BMC Systems Biology 3: 100. 19804630 10.1186/1752-0509-3-100 PMC2764635

[34] Huang S, Ren X, Wang L, Zhang L, Wu X (2011) Lung-cancer chemoprevention by induction of synthetic lethality in mutant KRAS premalignant cells in vitro and in vivo. Cancer Prevention Research 4: 666. 21543344 10.1158/1940-6207.CAPR-10-0235

[35] Zhang Z, Stiegler A, Boggon T, Kobayashi S, Halmos B (2010) EGFR-mutated lung cancer: a paradigm of molecular oncology. Oncotarget 1: 497. 21165163 10.18632/oncotarget.186 PMC3001953

[36] da Cunha Santos G, Shepherd F, Tsao M (2011) EGFR mutations and lung cancer. Annual Review of Pathology: Mechanisms of Disease 6: 49–69. 10.1146/annurev-pathol-011110-130206 20887192

[37] Adjei A (2001) Blocking oncogenic Ras signaling for cancer therapy. Journal of the National Cancer Institute 93: 1062–1074. 11459867 10.1093/jnci/93.14.1062

[38] Marino S, Hogue I, Ray C, Kirschner D (2008) A methodology for performing global uncertainty and sensitivity analysis in systems biology. Journal of Theoretical Biology 254: 178–196. 18572196 10.1016/j.jtbi.2008.04.011 PMC2570191

[39] Stone K, Mercer R, Gehr P, Stockstill B, Crapo J, et al (1992) Allometric relationships of cell numbers and size in the mammalian lung. American Journal of Respiratory Cell and Molecular Biology 6: 235. 1540387 10.1165/ajrcmb/6.2.235

[40] Fujioka A, Terai K, Itoh R, Aoki K, Nakamura T, et al (2006) Dynamics of the Ras/ERK MAPK cascade as monitored by fluorescent probes. Journal of Biological Chemistry 281: 8917–8926. 16418172 10.1074/jbc.M509344200

[41] Rudolph C, Adam G, Simm A (1999) Determination of copy number of c-Myc protein per cell by quantitative western blotting. Analytical Biochemistry 269: 66–71. 10094776 10.1006/abio.1999.3095

[42] Luscher B, Eisenman R (1988) c-myc and c-myb protein degradation: effect of metabolic inhibitors and heat shock. Molecular and Cellular Biology 8: 2504. 3043180 10.1128/mcb.8.6.2504-2512.1988 PMC363451

[43] Lim L, Lau N, Weinstein E, Abdelhakim A, Yekta S, et al (2003) The microRNAs of Caenorhabditis elegans. Genes & Development 17: 991. 12672692 10.1101/gad.1074403 PMC196042

[44] Liang Y, Ridzon D, Wong L, Chen C (2007) Characterization of microRNA expression profiles in normal human tissues. BMC Genomics 8: 166. 17565689 10.1186/1471-2164-8-166 PMC1904203

[45] Sethi P, Lukiw W (2009) Micro-RNA abundance and stability in human brain: specific alterations in Alzheimer's disease temporal lobe neocortex. Neuroscience Letters 459: 100–104. 19406203 10.1016/j.neulet.2009.04.052

[46] Iliopoulos D, Hirsch H, Struhl K (2009) An epigenetic switch involving NF-κ B, Lin28, let-7 microRNA, and IL6 links inflammation to cell transformation. Cell 139: 693–706. 19878981 10.1016/j.cell.2009.10.014 PMC2783826

[47] Chaplain M (2011) Multiscale mathematical modelling in biology and medicine. IMA Journal of Applied Mathematics 76: 371.

[48] Fujita Y, Krause G, Scheffner M, Zechner D, Leddy H, et al (2002) Hakai, a c-Cbl-like protein, ubiquitinates and induces endocytosis of the E-cadherin complex. Nature Cell Biology 4: 222–231. 11836526 10.1038/ncb758

[49] Safranek J, Pesta M, Holubec L, Kulda V, Dreslerova J, et al (2009) Expression of MMP-7, MMP-9, TIMP-1 and TIMP-2 mRNA in lung tissue of patients with non-small cell lung cancer (NSCLC) and benign pulmonary disease. Anticancer Research 29: 2513. 19596921

[50] Hopkins S, Henderson A, Levin D, Yamada K, Arai T, et al (2007) Vertical gradients in regional lung density and perfusion in the supine human lung: the Slinky effect. Journal of Applied Physiology 103: 240–248. 17395757 10.1152/japplphysiol.01289.2006 PMC2399899

[51] Akool E, Kleinert H, Hamada F, Abdelwahab M, Forstermann U, et al (2003) Nitric oxide increases the decay of matrix metalloproteinase 9 mRNA by inhibiting the expression of mRNA-stabilizing factor HuR. Molecular and Cellular Biology 23: 4901. 12832476 10.1128/MCB.23.14.4901-4916.2003 PMC162218

