# Supplementary material for: A Mathematical Model for MicroRNA in Lung Cancer
Source: PLoS One. 2013 Jan 24;8(1):e53663. doi: 10.1371/journal.pone.0053663 (PMC3554769; doi:10.1371/journal.pone.0053663)
Supplement: Material S1 — Experimental results of miR-9 in lung tumor tissues. Experimental results using quantitative reverse transcription polymerase chain reaction and in situ hybridization for miR-9 are provided. (PDF) [file pone.0053663.s001.pdf]

Supplementary Material S1
A mathematical model for microRNA in lung cancer

Hye-Won Kang[1,#], Melissa Crawford[2], Muller Fabbri[3], Gerard Nuovo[2], Michela Garofalo[3], S. Patrick Nana-Sinkam[2,#,*], Avner Friedman[1,4,#]

1 Mathematical Biosciences Institute, Ohio State University, Columbus, OH, USA

2 Davis Heart and Lung Research Institute, Ohio State University, Columbus, OH, USA

3 Department of Molecular Virology, Immunology and Medical Genetics, Ohio State University, Columbus, OH, USA

4 Department of Mathematics, Ohio State University, Columbus, OH, USA

# **Authors contributed equally to this manuscript.**

∗ **E-mail: Patrick.Nana-Sinkam@osumc.edu**

1 Experimental results

1.1 Quantitative Reverse Transcription Polymerase Chain Reaction

We performed independent assays by using QRT-PCR for miR-9 (Assay ID 000583, UCUUUGGUUAUCUAGCUGUAUGA, Applied Biosystems) on both tumor and adjacent uninvolved lung from 30 cases of NSCLC, which are typical cases out of 130 cases. All RT-PCR experiments were performed in three independent experiments conducted in triplicate on all study samples. Data were presented relative to RNU48 for microRNA based on calculations of $2^{-\Delta Ct}$. Statistical significance was defined as $p < 0.05$ as mea-

sured by Students t-test.

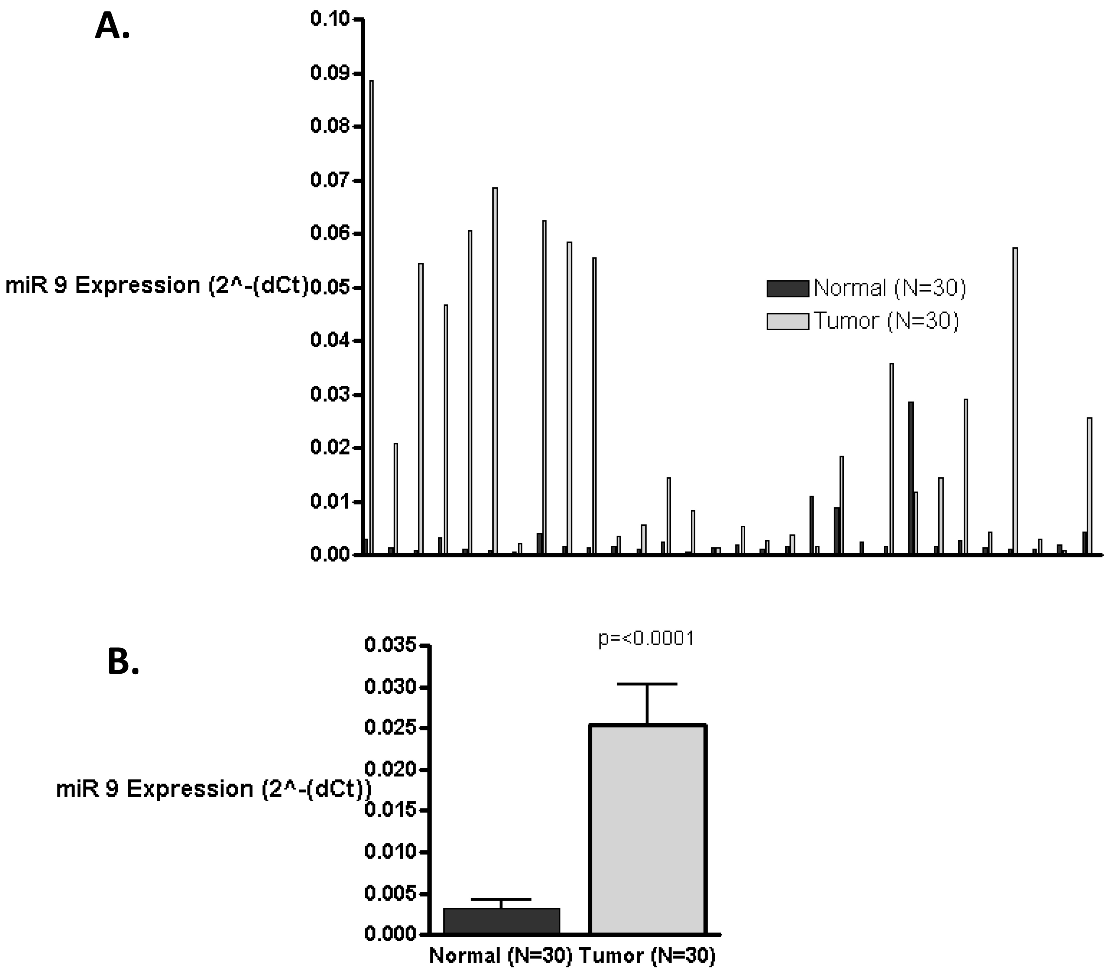

Figure S1. miR-9 expression in NSCLC. A. miR-9 expression by qRT-PCR in individual NSCLC and adjacent uninvolved lung (N=30). B. Increased miR-9 in group of NSCLC compared to adjacent uninvolved lung.

1.2 In Situ Hybridization for miR-9

In situ hybridization for miR-9 was performed as previously described [1]. We obtained a Locked nucleic acid (LNA)-modified probe with 5 digoxigenin labeling for miR-9 (Exiqon). Following protease digestion $2\,pmoles/L$ of the probe was hybridized to NSCLC and adjacent uninvolved tissue sections for 15 hours, then washed. The probe was visualized after nuclear fast red counterstain.

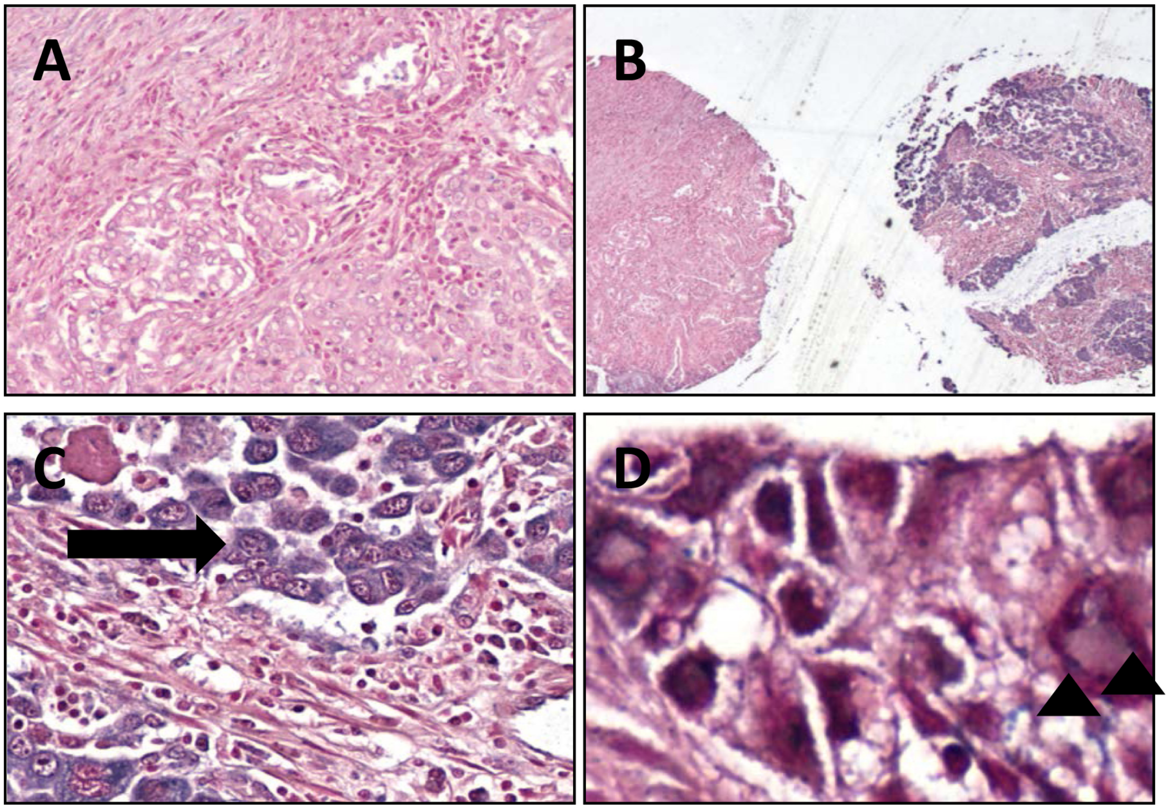

Figure S2. In situ hybridization of miR-9 in NSCLC: A. Scrambled miR demonstrating no signal in NSCLC. B. miR-9 in case of NSCLC compared to adjacent normal lung. C. Higher magnification of cytosolic staining for miR-9 (arrow). D. miR-9 (arrowheads).

References

1. Nuovo G, Elton T, Nana-Sinkam P, Volinia S, Croce C, et al. (2009) A methodology for the combined in situ analyses of the precursor and mature forms of microRNAs and correlation with their putative targets. Nature Protocols 4: 107–115.
